# Public Approval of Exception From Informed Consent in Emergency Clinical Trials: A Systematic Review of Community Consultation Surveys

**DOI:** 10.1001/jamanetworkopen.2019.7591

**Published:** 2019-07-24

**Authors:** William B. Feldman, Spencer P. Hey, Jessica M. Franklin, Aaron S. Kesselheim

**Affiliations:** 1 Division of Pulmonary and Critical Care Medicine, Department of Medicine, Brigham and Women’s Hospital, Harvard Medical School, Boston, Massachusetts; 2 Program On Regulation, Therapeutics, And Law, Division of Pharmacoepidemiology and Pharmacoeconomics, Department of Medicine, Brigham and Women’s Hospital, Harvard Medical School, Boston, Massachusetts; 3 Harvard Medical School Center for Bioethics, Boston, Massachusetts

## Abstract

**Importance:**

The US Food and Drug Administration (FDA) created the exception from informed consent (EFIC) pathway in 1996 to allow some emergency trials to enroll patients without informed consent. To protect individual autonomy and preserve public trust, the FDA requires that EFIC trial investigators consult with community members before a trial may begin.

**Objectives:**

To analyze data from surveys conducted as part of community consultation ahead of EFIC trials and assess levels of public approval.

**Data Sources:**

All trials granted an EFIC must submit documentation of compliance with EFIC regulations to a publicly available docket at the FDA. Submissions between November 1, 1996, and October 23, 2017, were reviewed.

**Study Selection:**

Trials with survey data were included.

**Data Extraction and Synthesis:**

Data were extracted between January 2018 and June 2018 and were analyzed between June 2018 and August 2018. The quality and validity of data were assessed according to Preferred Reporting Items for Systematic Reviews and Meta-analyses (PRISMA) guidelines. A random-effects metaregression was used to assess the association of demographic characteristics with EFIC approval.

**Main Outcomes and Measures:**

The primary study outcome was EFIC approval.

**Results:**

The FDA docket contained 15 958 pages of material with survey data for 42 448 individuals submitted by 27 trials. Public approval of EFIC varied by question type, with more people willing to approve initiation of EFIC trials in their community (86.5%) than personal enrollment (73.0%), enrollment of a family member (68.6%), or the principle of enrollment without consent (58.4%) (*P* < .001 for all comparisons). In the United States, African American individuals made up 29.3% of those enrolled in EFIC trials that reported data on race (5064 of 17 302) but only 16.7% of those surveyed as part of community consultation. In the United States and Canada, men made up 42.9% of the surveyed population but 65.6% of those eventually enrolled in EFIC trials (29 961 of 45 694). Groups surveyed with higher proportions of African American and male respondents had lower rates of EFIC approval.

**Conclusions and Relevance:**

Public approval of EFIC trials varied by question type and by the respondents’ reported race and sex. The demographic characteristics of those surveyed did not match the demographic characteristics of EFIC enrollees. The FDA could strengthen community consultation by standardizing survey instruments and reporting, requiring broader inclusion of African American and male respondents, clarifying the function of surveys in the development and modification of trial protocols, and building more public consensus around the acceptable use of EFIC.

## Key Points

**Question:**

How does the public view emergency research conducted with an exception from informed consent (EFIC)?

**Findings:**

In this systematic review of survey data from 27 emergency clinical trials with responses from 42 448 individuals submitted by EFIC trial organizers to the US Food and Drug Administration, public attitudes regarding EFIC varied: 58.4% approved of EFIC in principle, 68.6% approved of family-member enrollment, 73.0% approved of personal enrollment, and 86.5% approved of community inclusion. Groups surveyed with higher proportions of African American and male respondents had lower rates of EFIC approval, and these groups were underrepresented in surveys relative to their enrollment in EFIC trials.

**Meaning:**

The US Food and Drug Administration should aim to build greater public consensus around the appropriate use of EFIC.

## Teaser

This systematic review analyzes data from surveys conducted as part of community consultation to assess levels of public approval of trials conducted with an exception from informed consent.

## Introduction

In 1996, the Food and Drug Administration (FDA) created the exception from informed consent (EFIC) pathway for emergency clinical research. This pathway allows investigators to enroll patients without consent from the patient, their family, or their legally authorized representatives. To qualify for an EFIC, trials must be aimed at life-threatening emergencies with unproven or unsatisfactory treatments requiring intervention within a therapeutic window that is too narrow for prospective informed consent.^1^ The FDA has granted more than 40 EFICs during the past 2 decades, and these trials have enrolled more than 45 000 patients.^2^ Exception from informed consent trials have tested interventions for an array of conditions, including cardiac arrest, hemorrhagic shock, traumatic brain injury, status epilepticus, ischemic stroke, respiratory failure, and acute coronary syndrome.^2,3^ These trials have yielded useful clinical discoveries but also exposed patients to considerable risks.^2^

Bypassing prospective informed consent in the EFIC pathway presents ethical challenges for how to respect the autonomy of enrollees and safeguard public trust in the research enterprise. To address these challenges, the FDA mandates that EFIC investigators institute additional protections when initiating and conducting trials. A key protection is community consultation. Prior to initiating trials, investigators must disseminate information about their research and solicit feedback from community members, defined as “representatives of the communities in which the clinical investigation will be conducted and from which the subjects will be drawn.”^1^ Most investigators have relied, at least in part, on surveys to fulfill this requirement.^4^

The FDA has not specified what level of community approval in surveys is appropriate nor what role surveys should play in deliberations by institutional review boards charged with local approval and direct oversight of EFIC trials. However, the surveys conducted by trial investigators provide insight into the EFIC trial consent process, including the characteristics of the community members whom investigators reach before conducting EFIC trials and how attitudes regarding EFIC vary. The characteristics and findings of EFIC trial surveys are particularly important to assess because EFIC trial enrollment is marked by demographic asymmetry: a 2018 systematic review of EFIC trials^2^ found that, among 23 833 people enrolled in EFIC trials at US sites, race data were available for 17 302 (72.3%). African American individuals made up 29.3% of participants (5064 participants), while they represented 13.4% of the US population in 2018.^5^ Men made up 65.6% of enrollees overall (29 961 of 45 694).^2^ Since the FDA has emphasized the importance of consulting those most likely to be affected by EFIC trials, it is vital to understand whether surveys are reaching the community members most likely to be enrolled and how these populations and others respond to the surveys.^6^

There is limited literature on community consultation surveys in EFIC trials. Several trials have published survey data from 1 or more sites, but these studies capture only isolated snapshots of community attitudes regarding EFIC.^7,8,9,10,11,12,13,14,15,16,17,18^ To our knowledge, the largest systematic review of EFIC survey data to date examined 9 trials with 9036 respondents but included only published data and did not quantitatively assess the demographic characteristics of those surveyed.^19^ To comprehensively evaluate public attitudes regarding EFIC trials and avoid publication bias, we conducted a systematic review of all EFIC survey data submitted directly to the FDA since initiation of the EFIC pathway in November 1996 through October 2017. We characterized the number of surveys conducted per trial, the demographic characteristics of the surveyed population, and the differences in EFIC approval by question type (ie, questions about personal enrollment, enrollment of family members, participation of the community, or the principle of EFIC), survey type (ie, random-digit dialing or convenience sampling), race, and sex. A clearer understanding of the population surveyed and attitudes elicited is crucial not only to guide future EFIC investigators in this area but also to help regulators and ethicists evaluate the community consultation process.

## Methods

### Data Source and Search

This systematic review was completed in accordance with the Preferred Reporting Items for Systematic Reviews and Meta-analyses (PRISMA) reporting guideline (Figure).^20^ The FDA requires that trial investigators submit evidence of public disclosure to FDA docket 95S-0158. Public disclosure is distinct from community consultation in that the former requires investigators to raise awareness about trials while the latter requires obtaining feedback (eg, through surveys). While trials must meet both requirements, the FDA asks only that documentation of public disclosure be submitted to the docket.^6^ Nevertheless, most trials submit evidence of both community consultation and public disclosure.^4^ The docket is publicly available, and therefore, institutional review board approval was not required for this study. The docket was acquired via an in-person request and contained all survey data submitted by trial investigators from November 1, 1996, to October 23, 2017. There were 15 958 pages of material in the FDA docket, including 177 official documents and 289 draft documents (ie, documents that have not yet been sorted and categorized by the FDA but remain part of the public record).

**Figure.**
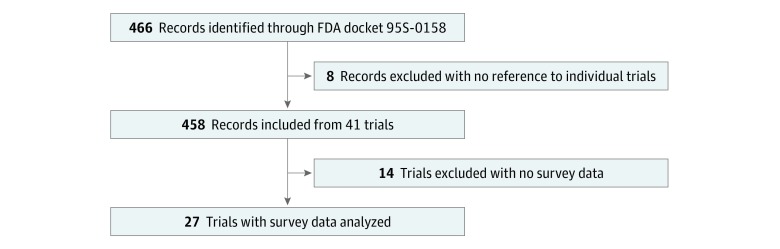
Preferred Reporting Items for Systematic Review and Meta-analyses Flow Diagram FDA indicates Food and Drug Administration.

### Study Selection

All documents were sorted by trial. Each document was then reviewed by one of us (W.B.F.) to look for survey data (eAppendix in the Supplement).

### Question and Answer Types

Questions from surveys were divided into 4 categories, which follow those used in previous studies^19^: (1) personal approval, ie, whether respondents would be willing to be enrolled in the EFIC trial in question; (2) family approval, ie, whether respondents would be willing for a family member to be enrolled in the EFIC trial in question; (3) community approval, ie, whether respondents would be willing for the EFIC trial in question to be conducted in their community; and (4) general approval, ie, whether respondents endorsed exceptions from informed consent in principle.

Survey answers were categorized as yes, no, or not sure/no answer. When surveys were conducted with a Likert scale, strongly agree and agree were counted yes, disagree and strongly disagree were counted no, and neutral was counted not sure/no answer.

### Data Extraction

Data were extracted by one of us (W.B.F.). Data consisted of the number of surveys per trial, sex of respondents, race of respondents, survey location (ie, United States or Canada), question type (ie, personal, family, community, or general), answer (ie, yes, no, or not sure/no answer), and sampling strategy (ie, random or convenience sampling).

### Statistical Analysis

Statistical analysis was conducted in R version 3.5.1 (The R Foundation). *P* values for differences in proportions were calculated using the 2-proportion *z* test. Two-tailed *P *values less than .05 were considered significant. To examine the association of demographic characteristics with approval rates by question type (personal, family, community, general), we fit a separate random-effects metaregression model for the proportion of patients answering yes to each question. Factors associated with this outcome in the metaregression included the proportion of African American individuals, the proportion of men, and the sampling strategy. The metaregression model was fit only for surveys with data on all 3 factors and the outcome of interest.

## Results

### Survey Submissions by Trial

Among the 41 trials that had been granted an EFIC and submitted data to the FDA by October 2017,^21,22,23,24,25,26,27,28,29,30,31,32,33,34,35,36,37,38,39,40,41,42,43,44,45,46,47,48,49,50,51,52,53,54,55,56,57,58,59,60,61^ 27 (65.9%) submitted survey data (Table 1 and Table 2). As part of these trials, investigators surveyed 42 448 individuals, some by random-digit dialing (17 342 [40.9%]) and the rest by convenience sampling in the community (25 106 [59.1%]).

**Table 1.** Surveys Submitted to the FDA by Trials Granted an Exception From Informed Consent

Trial	Year Material Was First Received by FDA	Random Sample Survey Responses, No.^a^	Convenience Sample Survey Responses, No.^a^
Clinical Investigation of the VEST-CPR System in Adults^21^	1997	0	25
Diaspirin Cross-Linked Hemoglobin (DCLhb) in the Treatment of Severe Traumatic Hemorrhagic Shock: A Randomized Controlled Efficacy Trial^22^	1997	0	0
Phase 2 Study of LeukArrest (ICOS anti-Cd11/Cd18 mAb) in Trauma-Induced Hemorrhagic Shock^23^	1998	1009	0
Randomized Clinical Trial of Magnesium, Diazepam, or Both After Out-of-Hospital Cardiac Arrest^24^	1998	35	13
Mechanical Thrombectomy for Acute Ischemic Stroke: Final Results of the Multi MERCI Trial^25^	2002	0	372
Clinical Evaluation of an Inspiratory Impedance Threshold Device During Standard Cardiopulmonary Resuscitation in Patients With Out-of-Hospital Cardiac Arrest^26^	2002	0	0
Public-Access Defibrillation and Survival After Out-of-Hospital Cardiac Arrest^27^	2003	0	0
Usefulness of Vasopressin Administered With Epinephrine During Out-of-Hospital Cardiac Arrest^28^	2003	0	0
Human Polymerized Hemoglobin for the Treatment of Hemorrhagic Shock When Blood is Unavailable: The USA Multicenter Trial^29^	2004	500	2493
Hypertonic Resuscitation of Hypovolemic Shock After Blunt Trauma: A Randomized Controlled Trial^30^	2004	500	0
Impact of Low-Dose Vasopressin on Trauma Outcome: Prospective Randomized Study^31^	2006	0	6
Treatment of Ventricular Tachyarrhythmias Refractory to Shock With Beta Blockers: The SHOCK and BLOCK Trial^32^	2006	0	0
Out-of-Hospital Administration of Intravenous Glucose-Insulin-Potassium in Patients With Suspected Acute Coronary Syndromes: The IMMEDIATE Randomized Controlled Trial^33^	2007	0	0
Out-of-Hospital Hypertonic Resuscitation Following Severe Traumatic Brain Injury: A Randomized Controlled Trial^34^	2007	3547	350
Out-of-Hospital Hypertonic Resuscitation After Traumatic Hypovolemic Shock: A Randomized, Placebo-Controlled Trial^35^	2007	3547	350
A Trial of an Impedance Threshold Device in Out-of-Hospital Cardiac Arrest^36^	2007	712	6
Early vs Later Rhythm Analysis in Patients With Out-of-Hospital Cardiac Arrest^37^	2007	712	6
Effect of Erythropoietin and Transfusion Threshold on Neurological Recovery After Traumatic Brain Injury^38^	2007	0	295
Very Early Hypothermia Induction in Patients With Severe Brain Injury (the National Acute Brain Injury Study: Hypothermia II): A Randomized Trial^39^	2008	0	957
Vasopressin Rescue for In-Pediatric Intensive Care Unit Cardiopulmonary Arrest Refractory to Initial Epinephrine Dosing: A Prospective Feasibility Pilot Trial^40^	2008	0	0
A Trial of Imaging Selection and Endovascular Treatment for Ischemic Stroke^41^	2009	0	0
CPR Quality Improvement During In-Hospital Cardiac Arrest Using a Real-Time Audiovisual Feedback System^42^	2009	0	0
Standard Cardiopulmonary Resuscitation vs Active Compression-Decompression Cardiopulmonary Resuscitation With Augmentation of Negative Intrathoracic Pressure for Out-of-Hospital Cardiac Arrest: A Randomized Trial^43^	2009	0	0
Effect of Prehospital Induction of Mild Hypothermia on Survival and Neurological Status Among Adults With Cardiac Arrest: A Randomized Clinical Trial^44^	2009	217	217
Comparison of Standard CPR vs CPR With an Intrathoracic Pressure Regulator vs Active Compression Decompression CPR Plus an Impedance Threshold Device During Out-of-Hospital Cardiac Arrest^45^	2011	0	139
AVERT Shock: Arginine Vasopressin During the Early Resuscitation of Traumatic Shock^46^	2012	0	309
Amiodarone, Lidocaine, or Placebo in Out-of-Hospital Cardiac Arrest^47^^b^	2012	2507	322
Trial of Continuous or Interrupted Chest Compressions During CPR^48^^b^	2012	503	0
Intramuscular vs Intravenous Therapy for Prehospital Status Epilepticus^49^	2012	1003	5953
Very Early Administration of Progesterone for Acute Traumatic Brain Injury^50^	2012	1592	5861
Lorazepam vs Diazepam for Pediatric Status Epilepticus: A Randomized Clinical Trial^51^	2012	508	0
A Controlled Resuscitation Strategy is Feasible and Safe in Hypotensive Trauma Patients: Results of a Prospective Randomized Pilot Trial^52^	2012	1509	360
Transfusion of Plasma, Platelets, and Red Blood Cells in a 1:1:1 vs a 1:1:2 Ratio and Mortality in Patients With Severe Trauma: The PROPPR Randomized Clinical Trial^53^	2012	1752	356
A Randomized, Double-Blind, Placebo-Controlled, Dose-Escalation Study of NNZ-2566 in Patients With Traumatic Brain Injury^54^	2012	0	0
A Randomized Comparative Multicenter, Open Label, Non-Inferiority Study, to Compare the SolidAIRity Airway Stabilization System’s Ability to Prevent Unplanned Extubation Relative to Standard of Care in Critically Ill or Injured Subjects Requiring Emergency Department or Intensive Care Unit Oral Intubation for Airway Management and Admission to the ICU^55^	2014	0	225
Prehospital Plasma During Air Medical Transport in Trauma Patients at Risk for Hemorrhagic Shock^56^	2014	0	0
Prehospital Tranexamic Acid Use for Traumatic Brain Injury^57^	2015	1000	238
Ketamine vs Etomidate for Sedation of Emergency Department Patients During Rapid Sequence Intubation^58^	2015	0	0
Effect of a Strategy of Initial Laryngeal Tube Insertion vs Endotracheal Intubation on 72-hour Survival in Adults With Out-of-Hospital Cardiac Arrest: A Randomized Clinical Trial^59^	2016	703	137
A Multicenter, Randomized, Blinded, Comparative Effectiveness Study of Fosphenytoin, Valproic Acid, or Levetiracetam in the Emergency Department Treatment of Patients With Benzodiazepine-Refractory Status Epilepticus^60^	2016	750	6478
Study of Tranexamic Acid During Air and Ground Medical Prehospital Transport Trial for Trauma Patients at Risk of Hemorrhage (STAAMP Trial): Phase III Multicenter, Prospective, Randomized, Double-Blind, Interventional Trial^61^	2017	0	0

Abbreviations: FDA, Food and Drug Administration; ICU, intensive care unit.

^a^ While a total of 48 074 surveys are recorded in this Table (22 606 by random sampling and 25 468 by convenience sampling), there were 5626 overlapping surveys (5264 by random sampling and 362 by convenience sampling). The number of individuals who generated the surveys in this table is therefore 42 448 (17 342 by random sampling and 25 106 by convenience sampling). See notes for Table 2 about the 3345 patients across 3 trials who were randomly sampled but who are grouped under the “convenience sampling” category for demographic characteristic analysis (because the authors did not disaggregate these individuals in reporting the much larger number of individuals surveyed by convenience sampling). Here, these 3345 individuals are grouped under “random sampling.”

^b^ Three groups of respondents (1254 individuals) were asked about 2 trials,^47,48^ but the docket only provided survey results from 1 of the trials.^47^

**Table 2.** Demographic Characteristics of the Surveyed Population in Data Submitted to the Food and Drug Administration by Trials Granted an Exception From Informed Consent

Characteristic	No. (%)		
	Random Sampling (n = 13 997)^a^	Convenience Sampling (n = 28 451)	Total (n = 42 448)
**Sex**			
Data available			
Unweighted data, No.	3021	24 182	27 203
Male	1086 (35.9)	9971 (41.2)	11 057 (40.6)
Female	1935 (64.1)	14 211 (58.8)	16 146 (59.4)
Weighted data, No.	9971	0	9971
Male	4875 (48.9)	0	4875 (48.9)
Female	5096 (51.1)	0	5096 (51.1)
All data, No.	12 992	24 182	37 174
Male	5961 (45.9)	9971 (41.2)	15 932 (42.9)
Female	7031 (54.1)	14 211 (58.8)	21 242 (57.1)
Data unavailable, No.	1005	4269	5274
Queried, no response	0	82 (1.9)	82 (1.6)
Queried, not provided	505 (50.2)	161 (3.8)	666 (12.6)
Not queried	0	0	0
Not provided, unclear if queried	500 (49.8)	4026 (94.3)	4526 (85.8)
**Race**^b^			
Data available, No.	9216	24 312	33 528
White	7393 (80.2)	17 396 (71.6)	24 789 (73.9)
African American	1267 (13.7)	4246 (17.5)	5513 (16.4)
Other	556 (6.0)	2670 (11.0)	3226 (9.6)
Data unavailable, No.	4802	4144	8946
Refused or not known	245 (5.1)	210 (5.1)	455 (5.1)
Not queried	1493 (31.1)	134 (3.2)	1627 (18.2)
Queried, not provided	1005 (20.9)	337 (8.1)	1342 (15.0)
Not provided, unclear if queried	2059 (42.9)	3463 (83.6)	5522 (61.7)
**Survey Location**			
United States	12 343 (88.2)	28 421 (99.9)	40 764 (96.0)
Canada	1654 (11.8)	30 (0.1)	1684 (4.0)

^a^ Three trials contained survey data from random-digit dialing that were aggregated with the data from convenience sampling and not provided separately: 1003 by random sampling out of 6956 surveyed in 1 trial^49^; 1592 by random sampling out of 7453 surveyed in another^50^; 750 by random sampling out of 7228 surveyed in the third.^60^ Because the data could not be disaggregated and most individuals were queried by convenience sampling, the data from these 3 trials were grouped under the category of convenience sampling.

^b^ No data were weighted by race. In 3 trials, investigators provided the sex and race (by percentage) of individuals who participated in community consultation,^49,50,60^ and it is not clear if these percentages refer to those who completed surveys or all who participated in community consultation. For the purposes of analysis, we have used these percentages as reflecting those who completed surveys. In 6 surveys (conducted in 4 different trials), individuals could select more than 1 race, and investigators reported the aggregated data of all races selected: (1) 100 surveyed, 102 included^44^; (2) 322 surveyed, 327 included^47^; (3) 502 surveyed, 504 included^47^; (4) 400 surveyed, 410 included^57^; (5) 400 surveyed, 406 included^57^; (6) 400 surveyed, 401 included.^59^ As a result, the number of individuals categorized by race (14 018 individuals by random sampling and 28 456 by convenience sampling for a total of 42 474) is greater than the total number of individuals surveyed (13 997 individuals by random sampling and 28 451 by convenience sampling for a total of 42 448).

Investigators asked some of these 42 448 individuals about more than 1 trial in a single questionnaire. In particular, there were 3 groups who answered questions about multiple trials: 3897 individuals answered questions about 2 trauma trials examining hypertonic saline,^34,35^ and a subset of this group (508 individuals) also answered questions about 2 out-of-hospital cardiac arrest trials.^36,37^ A second group of 210 individuals answered questions exclusively about the 2 previously mentioned out-of-hospital cardiac arrest trials,^36,37^ and a third group of 503 individuals answered questions about 2 different out-of-hospital cardiac arrest trials.^47,48^ To ensure that questionnaires asking about multiple trials counted for each trial when quantifying submissions to the FDA, we separated questionnaires by trial (eg, so that a single questionnaire asking about 2 trials counted as 2 distinct surveys). This gave a total of 48 074 surveys (22 606 by random sampling and 25 468 by convenience sampling). The mean (SD [range]) number of surveys submitted per trial was 1781 (2257 [6-7453]). The mean (SD [range]) number of surveys submitted with random sampling per trial was 1214 (1040 [35-3547]), while the mean (SD [range]) number of surveys submitted with convenience sampling per trial was 1107 (2043 [6-6478]).

### Demographic Characteristics

Among the 42 448 individuals surveyed, data on sex were available for 37 174 (87.6%) (Table 2). Surveys for 27 203 individuals (73.2%) were unweighted by sex, and these surveys consisted of 11 057 men (40.6%) and 16 146 women (59.4%). Surveys for 9971 individuals (26.8%) were weighted by sex; after weighting, these surveys consisted of 4875 men (48.9%) and 5096 women (51.1%). Of 9971 weighted surveys, unweighted sex was also provided for 3507 individuals (1485 [42.3%] men and 2022 [57.7%] women). For the purposes of defining a cohort for analysis, the weighted data on sex were used for these individuals because the survey responses were based on weighted data. Therefore, in the total surveyed sample with sex data available (9971 weighted and 27 203 unweighted), there were 15 932 men (42.9%) and 21 242 women (57.1%).

Data on race were available for 33 528 individuals (78.9%). Of these individuals, 24 789 (73.9%) were white, 5513 (16.4%) were African American, and 3226 (9.6%) were of another race. No surveys were weighted by race.

Most individuals (40 764 [96.0%]) were surveyed in the United States, while a minority (1684 [4.0%]) were surveyed in Canada. Among those surveyed in the United States with data on race available (32 898 [80.7%]), 24 296 individuals (73.9%) were white, 5487 (16.7%) African American, and 3115 (9.5%) of another race.

### Approval by Question Type

Surveyed individuals demonstrated substantial variability in their approval of EFIC depending on the question category. Overall, 9923 (58.4%) approved of EFIC in principle, 4407 (68.6%) approved of family member enrollment, 25 295 (73.0%) approved of personal enrollment, and 12 340 (86.5%) approved of community inclusion (Table 3). (Questions by type are listed in eTables 1-4 in the Supplement.) Owing to large sample sizes, all pairwise comparisons of these question types were statistically significant (*P* < .001 for all comparisons).

**Table 3.** Attitudes Regarding Exception From Informed Consent

Survey Responses	No. (%)			*P* Value
	Random Sampling	Convenience Sampling	Total	
**Personal**				
Total, No.	13 994	20 677	34 671	NA
Approval	9798 (70.0)	15 497 (74.9)	25 295 (73.0)	<.001
Rejection	2944 (21.0)	2487 (12.0)	5431 (15.7)	
No answer/neutral	1252 (8.9)	2693 (13.0)	3945 (11.4)	
**Family Member**				
Total, No.	2317	4111	6428	NA
Approval	1699 (73.3)	2708 (65.9)	4407 (68.6)	<.001
Rejection	451 (19.5)	924 (22.5)	1375 (21.4)	
No answer/neutral	167 (7.2)	479 (11.7)	646 (10.0)	
**Community**				
Total, No.	5205	9062	14 267	NA
Approval	4194 (80.6)	8146 (89.9)	12 340 (86.5)	<.001
Rejection	483 (9.3)	609 (6.7)	1092 (7.7)	
No answer/neutral	528 (10.1)	307 (3.4)	835 (5.9)	
**General**				
Total, No.	5386	11 609	16 995	
Approval	3674 (68.2)	6249 (53.8)	9923 (58.4)	<.001
Rejection	1170 (21.7)	3039 (26.2)	4209 (24.8)	
No answer/neutral	542 (10.1)	2321 (20.0)	2863 (16.8)	

Abbreviation: NA, not applicable.

### Convenience vs Random Sampling

Individuals who were surveyed by convenience sampling were more likely than those surveyed by random sampling to approve of personal enrollment (15 497 [74.9%] vs 9798 [70.0%]; *P* < .001) and initiation of EFIC trials in their community (8146 [89.9%] vs 4194 [80.6%]; *P* < .001) (Table 3). However, they were less likely to approve of enrollment of a family member (2708 [65.9%] vs 1699 [73.3%]; *P* < .001) and to endorse the general principle of EFIC (6249 [53.8%] and 3674 [68.2%]; *P* < .001).

### Metaregression Model of Race and Sex

We could not directly assess whether race and sex were associated with individual approval because most data submitted to the FDA docket were aggregated and failed to include a breakdown of responses by race or sex. However, there have been a high number of aggregates submitted to the FDA during the last 2 decades, and many trials provided information about race and sex for each aggregate even when they did not provide information about survey responses broken down by race or sex. Therefore, we were able to answer the question: do aggregates with higher percentages of African American individuals or higher percentages of men tend to have higher rates of approval when controlling for sampling strategy (random vs convenience)?

A random-effects metaregression model revealed that aggregates with higher percentages of African American individuals and higher percentages of men tended to have lower rates of approval (Table 4). When respondents were asked about being personally enrolled in EFIC trials without consent, for every 10% increase in the percentage of men, there was a 16.0% reduction in the odds of approval (odds ratio [OR], 0.840; 95% CI, 0.739-0.955). When respondents were asked about having a family member enrolled in EFIC trials without consent, for every 10% increase in the percentage of African American individuals, there was an 18.0% reduction in the odds of approval (OR, 0.820; 95% CI, 0.717-0.937), and for every 10% increase in the percentage of men, there was a 16.2% reduction in the odds of approval (OR, 0.838; 95% CI, 0.713-0.984). When respondents were asked about initiation of trials in their communities, for every 10% increase in the percentage of African American individuals, there was a 13.0% reduction in the odds of approval (OR, 0.870; 95% CI, 0.790-0.958), and for every 10% increase in the percentage of men, there was an 11.2% reduction in the odds of approval (OR, 0.888; 95% CI, 0.792-0.996). When respondents were asked about the principle of EFIC in general, for every 10% increase in the percentage of African American individuals, there was a 29.7% reduction in the odds of approval (OR, 0.703; 95% CI, 0.570-0.866).

**Table 4.** Metaregression of Survey Data Submitted to the Food and Drug Administration by Trials Granted an Exception From Informed Consent

Metaregression Variable	OR (95% CI)^a^	*P* Value
**Personal: 85 Aggregates, 27 579 Participants**		
Intercept	5.827 (3.058-11.102)	<.001
African American race	0.914 (0.824-1.013)	.09
Male sex	0.840 (0.739-0.955)	.008
Convenience sampling	3.146 (2.170-4.561)	<.001
**Family: 58 Aggregates, 4003 Participants**		
Intercept	4.420 (1.368-14.280)	.01
African American race	0.820 (0.717-0.937)	.004
Male sex	0.838 (0.713-0.984)	.03
Convenience sampling	6.044 (1.973-18.517)	.002
**Community: 59 Aggregates, 12 371 Participants**		
Intercept	8.904 (5.016-15.804)	<.001
African American race	0.870 (0.790-0.958)	.004
Male sex	0.888 (0.792-0.996)	.04
Convenience sampling	2.465 (1.766-3.442)	<.001
**General: 16 Aggregates, 14 427 Participants**		
Intercept	4.325 (1.253-14.931)	.02
African American race	0.703 (0.570-0.866)	.001
Male sex	1.022 (0.810-1.290)	.86
Convenience sampling	0.576 (0.354-0.935)	.03

Abbreviation: OR, odds ratio.

^a^ Odds ratios are for a 10% difference in African American race and male sex.

## Discussion

Surveys have been an integral component of community consultation efforts required by the FDA in EFIC trials. Nearly two-thirds of trials submitted some form of survey data to the FDA docket probing community attitudes. Our evaluation of these survey responses found that most individuals were willing to approve initiation of trials in their community without prospective consent even though, paradoxically, only about half were willing to endorse the use of EFIC in principle. We also found that the demographic characteristics of those who were surveyed as part of community consultation did not reflect the demographic characteristics of those who were eventually enrolled in EFIC trials. African American individuals made up 16.7% of those surveyed in the United States but 29.3% of those eventually enrolled at US sites with data available (note that the percentage of African American individuals enrolled at Canadian sites is not known as most Canadian institutional review boards did not permit the collection of race data in EFIC trials).^2^ Men made up 42.9% of those surveyed overall but 65.6% of those eventually enrolled.^2^ Surveyed groups with higher proportions of African American individuals and men were also less supportive of EFIC.

Our review supports the key finding of a previous, more limited review of EFIC trial surveys,^19^ which showed strong approval of community inclusion, less approval for personal participation, and the least approval for EFIC in general. Some have hypothesized that individual or family support may be weaker than community support because people prefer not to make decisions on behalf of others and thereby deprive them of opportunities for novel treatments in clinical research.^19,62^ However, this hypothesis does not explain the gap between community approval and general approval, both of which entail judgments on behalf of others. The discrepancy between community and general approval has led some to advocate for placing more value on questions of personal enrollment.^19^

The discrepancy also underscores the importance of framing effects and question phrasing. There were important differences in how questions were posed across categories (ie, personal, family, community, and general), and such differences were also evident within categories. For example, the personal question was posed in a variety of ways, probing whether respondents would “want” an intervention, would “want to be entered into the study and possibly receive”^34,35,36,37^ an intervention, would “want to be enrolled into this type of study,”^24,29,34,35,53^ would “be willing to be part of this study,”^34,35,52,57,59^ would “accept being enrolled in this study,”^53^ or would “be okay with being included”^50,60^ (eTable 1 in the Supplement). In many cases, the same trial phrased a question 1 way in surveys conducted at 1 site and another way in surveys conducted at different sites. This heterogeneity highlights the need for validated survey instruments to gauge community attitudes, ideally instruments that are sanctioned by the FDA and used across trials. Such an approach would facilitate more meaningful comparisons of public attitudes in EFIC trials and enable institutional review boards and the FDA to identify outlier trials with low approval.

African American individuals were underrepresented both among those surveyed by random sampling (1267 of 9216 [13.7%] surveyed) and those surveyed by convenience sampling (4246 of 24 312 [17.5%] surveyed). The data generated by random sampling were from random-digit dialing surveys conducted in the geographic location of trial sites (as determined by county or zip code). There are several potential explanations for African American underrepresentation in random-digit dialing surveys: investigators may have oversampled or undersampled certain zip codes or counties relative to enrollment, contacted African American individuals less frequently, or received fewer responses from African American individuals. Alternatively, individuals who were eventually enrolled—who, in most cases, experienced either cardiac arrest, hemorrhagic shock, or traumatic brain injury—may have had a racial composition different from the composition of geographic communities from which these individuals were drawn. Identifying a population for random sampling to match the demographic characteristics of EFIC enrollees may be challenging. However, investigators notably chose not to weight survey responses by race, as was done for sex in some instances.

Convenience sampling also failed to capture a percentage of African American individuals to match EFIC enrollment. It is common among EFIC investigators and encouraged by the FDA to connect with specific groups in the community who are more likely to be enrolled (eg, motorcycle groups in studies of traumatic brain injury) or who may have particular sensitivities to the research in question (eg, Jehovah’s Witnesses in studies about blood products). Investigators in EFIC trials reported that they specifically reached out to African American groups in many cases. Yet, African American individuals were only slightly better represented in convenience sampling than in random sampling.

The unweighted data on sex constituted 73.2% of all data on sex and included low percentages of male respondents in random samples (1086 of 3021 individuals [35.9%]) and convenience samples (9971 of 24 182 individuals [41.2%]). The potential reasons for underrepresentation of men could mirror some of the potential reasons for underrepresentation of African American individuals: fewer contacts with men by telephone, lower response rates of those contacted, higher likelihood of experiencing the condition under investigation, and lower attendance at community events. To correct for these potential pitfalls, investigators weighted some data (26.8%) by sex, and men represented 48.9% of respondents in the weighted samples. While weighting may achieve fairer representation, investigators chose to weight sex according to percentages in the population as a whole rather than percentages of likely EFIC enrollees. The FDA has not issued guidance about whether or how to weight surveys, but weighting surveys according to percentages of likely enrollees appears to be more in keeping with the objectives of community consultation.

A challenge for analyzing the survey data submitted to the FDA is that the data are not presented in a standard format and are often aggregated with no accompanying raw data. Our metaregression attempted to assess how race and sex influence EFIC acceptance even in the absence of individual raw data, but this approach is less generalizable than a regression of individual survey responses. While we can affirm a group-level association of demographic characteristics with responses of those surveyed, we cannot surmise further about the attitudes of individual African American individuals or men. The hypotheses generated by our metaregression merit follow-up with further individual-level assessment of raw survey data. The FDA could facilitate such work by requiring that all future survey data be submitted according to an agreed-on format that includes individual-level responses. A key goal of community consultation is to promote public trust in EFIC trials, and more standardized reporting of community attitudes would help foster such trust.

The FDA could take further steps to build public trust by delineating the desired extent of community consultation, clarifying the steps that investigators should take in light of survey results, and requiring that investigators submit documentation of how trial protocols are amended, if at all, based on community concerns. Measuring the efficacy of community consultation—for example, through surveys after the completion of EFIC trials to gauge public awareness and attitudes regarding the research enterprise—may also help refine the process. The FDA might even consider launching a campaign of its own (separate from individual trials) to increase public awareness of EFIC, solicit feedback for improvement, and build consensus about the appropriate use of EFIC. The National Institutes of Health recently undertook such an effort^63^ in the wake of a US House of Representatives committee inquiry into the conduct of a National Institutes of Health–funded EFIC trial.^64^ After more than 2 decades of experience with community consultation in EFIC trials, efforts to improve this important EFIC-related activity are vital.

### Limitations

In addition to heterogeneous question phrasing and insufficient individual-level raw data, this study has other limitations. First, given the person-hours required to extract data from the 15 958-page FDA docket, a single author completed the data extraction. Second, while this study was more comprehensive than prior studies because of our reliance on the FDA docket, the docket itself is incomplete. For example, an EFIC trial conducted 2079 surveys that were published but not included in the FDA docket.^16,33^ One reason the docket is incomplete is that the FDA does not specifically require trials to submit evidence of community consultation, only of public disclosure.^6^ Third, while surveys with random sampling were all conducted by random-digit dialing, surveys categorized as convenience samples were conducted in a range of settings, from intimate community meetings with long descriptions of the proposed research to large community fairs with only short descriptions. Because investigators provided variable information to respondents before surveying them, comparisons of convenience surveys with each other (or with random-digit dialing surveys in which scripts were used) are subject to bias. We controlled for type of sampling approach (random vs convenience) in all metaregression analyses, but we cannot control for variation in approach within the subset of convenience samples. Moreover, the convenience samples themselves may not be representative of the broader population. Fourth, this review did not analyze several important variables that might influence EFIC approval, including demographic characteristics such as age, income, and education, and trial characteristics, such as the condition under investigation (eg, trauma, cardiac arrest). These variables could not be reliably compared across surveys. Fifth, this review examines only a single component of community consultation, surveys.

More work is needed to understand the gamut of community consultation activities and public disclosure activities that are documented and submitted to the FDA. One recent review of material submitted to the FDA docket 95S-0158^4^ attempted to catalog these activities. However, that review examined online material only (with 177 official documents containing 6998 pages) rather than the full docket available by in-person request (with 177 official documents and 289 draft documents containing 15 958 pages at the time of this review).

## Conclusions

Trial investigators have relied on surveys for the last 2 decades to probe attitudes regarding EFIC. These surveys reveal substantial variation in question type and survey type. They also show that African American individuals and men are underrepresented in surveys relative to their enrollment in EFIC trials and that groups with higher proportions of African American individuals and men support EFIC at lower rates. The community consultation process would be strengthened by the adoption of validated and standardized surveys and reporting, more clarity about the function of surveys in the development and modification of trial protocols, broader efforts to increase public agreement about the acceptable use of EFIC, and further attempts to either reach groups likely to be enrolled in EFIC trials or to weight data accordingly. These improvements and others could help foster public trust and ensure the integrity of the EFIC pathway.

## Notes

Supplement. **eAppendix.** Supplemental Methods **eTable 1. **Personal Questions **eTable 2. **Questions About Family Members **eTable 3. **Questions About Community **eTable 4. **Questions About Exceptions from Informed Consent in General **eReferences**
