# Supplementary material for: Public Approval of Exception From Informed Consent in Emergency Clinical Trials: A Systematic Review of Community Consultation Surveys
Source: JAMA Netw Open. 2019 Jul 24;2(7):e197591. doi: 10.1001/jamanetworkopen.2019.7591 (PMC6659147; doi:10.1001/jamanetworkopen.2019.7591)
Supplement: Supplement. — eAppendix. Supplemental Methods eTable 1. Personal Questions eTable 2. Questions About Family Members eTable 3. Questions About Community eTable 4. Questions About Exceptions from Informed Consent in General eReferences [file jamanetwopen-2-e197591-s001.pdf]

Supplementary Online Content

Feldman WB, Hey SP, Franklin JM, Kesselheim AS. Public approval of exception from informed consent in emergency clinical trials: a systematic review of community consultation surveys. *JAMA Netw Open*. 2019;2(7):e197591. doi:10.1001/jamanetworkopen.2019.7591

eAppendix. Supplemental Methods
eTable 1. Personal Questions
eTable 2. Questions About Family Members
eTable 3. Questions About Community
eTable 4. Questions About Exceptions from Informed Consent in General
eReferences

This supplementary material has been provided by the authors to give readers additional information about their work.

© 2019 Feldman WB et al. *JAMA Network Open.*

eAppendix. Supplemental Methods

<u>Study selection</u>

Reports from community meetings of verbal approval, nods, or hand-raising were excluded. We included all survey material submitted by trials when quantifying the number of surveys conducted (Table 1) and the demographics of those surveyed (Table 2). However, when analyzing responses by question-type (Table 3), we included only surveys with questions that could be classified into one of the 4 categories (personal, family, community, general).

<u>Data extraction</u>

Data were not collected about the age of respondents, because this was often reported as a categorical rather than continuous variable and could not be adequately compared across surveys. When only percentages were provided for the variables collected, we back-calculated raw numbers. When surveys were conducted in another language, we used the translation of questions provided by the trial investigators.

When trials submitted overlapping data from the same participants (e.g. individual surveys from participants along with summaries of surveys from the same participants), the data were counted only once and the dataset with more respondents was used if there was a discrepancy in the overlapping data (e.g. when fewer individual surveys were submitted than were included in summary data that trials submitted). We excluded survey data that could not be interpreted due to illegibility, incomplete or unclear labelling, or confusion about language.

© 2019 Feldman WB et al. *JAMA Network Open.*

All included questions (eTables 1 – 4) explicitly reference the absence of consent either in the question itself, in an accompanying question, or in a passage or presentation describing the study before the question. A small number of questions had more than one component; questions with both a personal and family component were counted as personal questions, and questions with both a community and general component were counted as general questions. In some instances, respondents were asked a personal question phrased prospectively (whether they would be willing to be enrolled in a study) and a personal question phrased retrospectively (whether they would be upset if they later learned that they had been enrolled in the study). We included prospective rather than retrospective personal questions. Otherwise, when a group of individuals was asked about more than one question that fit under the same category (for example, if a survey included two community questions), we only analyzed the question with more responses.

Disaggregated data

Most trialists submitted aggregated data to the FDA with survey responses that were not stratified by race or sex. However, some trialists provided stratified data. Survey responses for 2,323 people were stratified by race (out of 33,528 people for which data on race were available), while survey responses for 4,406 people were stratified by sex (out of 37,174 people for which data on sex were available). In many cases, trialists provided such stratified data only when an association was observed between race or sex and survey responses. Other trialists who tested for such associations and found none often reported only aggregated data. Thus, to avoid biasing our findings, we rely

© 2019 Feldman WB et al. *JAMA Network Open.*

exclusively on aggregated data for our metaregression even when the same data are available in disaggregated form.

Aggregated data

We identified 39 aggregates submitted by trialists from surveys conducted by random digit dialing (ranging in size from 5 individuals to 508 individuals) and 183 aggregates submitted by trialists from surveys conducted by convenience sampling (ranging in size from 1 individual to 7,315 individuals). Most of the data from both random and convenience sampling were submitted to the FDA as summaries (and these summaries defined each aggregate). However, some investigators submitted hard copies of the forms completed by respondents. We counted these by hand and grouped surveys together as aggregates that were completed at the same time and place (754 people, 56 aggregates).

© 2019 Feldman WB et al. *JAMA Network Open.*

eTable 1. Personal Questions*

Question	Trial	# Asked	Document
If you were severely injured and had a one in three chance of dying with standard treatment, would you want to be entered into the study and possibly receive this experimental treatment, even though you couldn't give consent?	HS-TBI[1]/ HS-Shock[2]	502	2-A1
	HS-TBI[1]/ HS-Shock[2]	504	102-A1
	HS-TBI[1]/ HS-Shock[2]	502	Draft 358-A1
	HS-TBI[1]/ HS-Shock[2]	9	102-A1
	HS-TBI[1]/ HS-Shock[2]	73	102-A1
	HS-TBI[1]/ HS-Shock[2]	50	102-A1
	HS-TBI[1]/ HS-Shock[2]	2	102-A1
	HS-TBI[1]/ HS-Shock[2]	1	Draft 359-A1
	HS-TBI[1]/ HS-Shock[2]	2	Draft 359-A1
	HS-TBI[1]/ HS-Shock[2]	3	Draft 359-A1
At any moment, we are all at risk of serious injury, especially in an automobile. If you were severely injured, such that you had a 25-50% chance of dying with standard treatment, would you want the experimental fluid given to you without written consent, knowing that it might improve your chance of survival or recovery, but that there is a risk of allergic reaction, which is less than 1:100,000, or other unexpected side effects?	HS-TBI[1]/ HS-Shock[2]	302	15-A1
If you were severely injured and had a 25 to 50% chance of dying with standard treatment, would you want this experimental fluid given to you without written consent, knowing that it might improve your chance for survival or recovery even with a risk of allergic reaction or other unexpected side effects?	HS-TBI[1]/ HS-Shock[2]	150	15-A1
At any moment, we are all at risk of serious injury, especially in an automobile. If you were severely injured, such that you had a 25-50% chance of dying with standard treatment, would you want this experimental fluid given to you without written consent, knowing that it might improve your chance of survival or recovery from head injury, but that there is a risk of allergic reaction or other unexpected side effects?	HS-TBI[1]/ HS-Shock[2]	305	20-A1
	HS-TBI[1]/ HS-Shock[2]	500	Draft 357-A1
	HS-TBI[1]/ HS-Shock[2]	505	Draft 366-A1
	HS-TBI[1]/ HS-Shock[2]	5	Draft 366-A1
If you were severely injured and had a one in three chance of not surviving with the standard treatment, would you want to have this experimental treatment given to you without consent?	HS-TBI[1]/ HS-Shock[2]	186	Draft 361-A1
If you were severely injured as just described, would you be willing to be part of this study without consent? This includes possibly being given the study fluid without your consent.	HS-TBI[1]/ HS-Shock[2]	86	Draft 361-A1
If you suffered a cardiac arrest and had less than a 10% chance of surviving with standard treatment, would you want to be	PRIMED[3,4]	502	2-A1
	PRIMED[3,4]	1	Draft 359-A1

© 2019 Feldman WB et al. *JAMA Network Open.*

Question	Trial	# Asked	Document
entered into the study and possibly receive the experimental treatments, even though you couldn't give consent?	PRIMED[3,4]	2	Draft 359-A1
	PRIMED[3,4]	3	Draft 359-A1
If you or a relative had an out-of-hospital cardiac arrest and had a very high chance of dying, would you want this experimental CPR treatment, even if no one yet knows if the treatment is better or worse than the way CPR is already done? Would you want this treatment knowing that it would be done without your consent?	PRIMED[3,4]	210	6
At any moment, we are all at risk of serious injury, especially in an automobile. If you were severely injured, such that you had a 25-50% chance of dying with standard treatment, would you want this experimental drug given to you without written consent, knowing that it might improve your chance for survival but that there is a risk of infection?	Hu23F2G[5]	501	39-A4
At any moment, we are all at risk of serious injury, especially in an automobile. If you were severely injured, such that you had a 25-50% chance of dying with standard treatment, would you want this newly developed drug given to you without written consent, knowing that it might improve your chance for survival but that there is a risk of infection?	Hu23F2G[5]	508	39-A6
At any moment, we are all at risk of serious injury, especially in an automobile. If you were severely injured and had a 17-56% chance of dying with the best available standard treatment, would you want this experimental fluid given to you knowing that it might improve your chance for survival, even though you did not give consent and there is a risk of allergic reaction or other unexpected side effects?	PolyHeme[6]	500	86
If you had a cardiac arrest and were treated by Seattle paramedics, would you want to be enrolled into this type of study?	Mg-Diazepam-Both[7]	35	101-A1
	Mg-Diazepam-Both[7]	6	101-A1
	Mg-Diazepam-Both[7]	7	101-A1
If you or your relative had an out-of-hospital cardiac arrest and had a very high chance of dying or were at risk of brain damage, would you want this experimental procedure of cooling the body, if you thought it might improve the chance of survival or recovery?	Prehospital Hypothermia[8]	100	106-A2
If you or your relative had an out-of-hospital cardiac arrest would you want to have this experimental cooling treatment even if no one yet knows if the treatment is better? Would you want to have this treatment knowing that it would be done without your consent?	Prehospital Hypothermia[8]	117	106-A3
If you were severely injured and had a one in three chance of dying would you be willing to be part of this study without first providing consent?	HYPO RESUS[9]	503	122-A1
	HYPO RESUS[9]	115	122-A1
	HYPO RESUS[9]	502	124-A1
	HYPO RESUS[9]	504	128-A1
Let's say you had a cardiac arrest and are unconscious. You receive CPR during which pressing on the chest either is or is	CCC[10]	503	114-A1

Question	Trial	# Asked	Document
not interrupted in order to give breaths. Is it acceptable to you to receive such treatment and be enrolled in this study even though you could not give consent beforehand?			
Let's say you were going to the grocery store and had a cardiac arrest and are unconscious. No family members are with you. Emergency medical staff arrive within a few minutes and administer the standard treatment which includes CPR and an electric shock to restart your heart. You are enrolled in this study and receive one of the medicine study treatments. Is it acceptable to you to be enrolled in this study even though you could not provide consent?	ALPS[11]	503	114-A1
Let's say you were going to the grocery store and had a cardiac arrest and are unconscious. No family members are with you. Emergency medical staff arrive within a few minutes and administer the standard treatment which includes CPR and an electric shock to restart your heart. You are also enrolled in this study and receive one of the three study solutions. Is it acceptable to you to be enrolled on this study even though you could not provide consent?	ALPS[11]	500	119-A1
	ALPS[11]	252	120-A1
	ALPS[11]	502	138-A1
	ALPS[11]	500	151-A1
Let's say you were going to the grocery store and had a cardiac arrest and are unconscious. No family members are with you. Emergency medical staff arrive within a few minutes and administer the standard treatment which includes CPR and an electric shock to restart your heart. You are also enrolled in this study and receive one of the three study solutions. Is it acceptable to be enrolled on this study even though you could not provide consent?	ALPS[11]	250	135-A1
If you were unconscious after a severe automobile accident and lost a large amount of blood, would you accept being enrolled in this study even though you could not provide consent?	PROPPR[12]	500	130-A2
	PROPPR[12]	252	136-A1
At any moment, we are all at risk of serious injury, especially in an automobile. If you were severely injured and it was determined that you would need blood products, would you find it acceptable to be enrolled in this study with an independent physician authorization and delayed informed consent?	PROPPR[12]	500	130-A3
…if you were critically injured and needed blood transfusions would you want to be in a study that tested two different combinations of blood products even if we don't know if one is better than the other? Would you want to be in this study if we could not ask you first? That is, if we enrolled you in the study without your consent?	PROPPR[12]	500	131-A1
If you experienced a severe brain injury would you be willing to be part of this study without first providing consent?	TXA[13]	400	157-A1
If you experienced a severe brain injury and had about a one in four chance of dying or one in three chance of experiencing decreased mental recovery would you be willing to be part of this study without first providing consent?	TXA[13]	400	158-A1
If you experienced a severe brain injury and had about a one in four chance of dying or one in three chance of experiencing decreased mental ability would you be willing to be part of this study without first providing consent?	TXA[13]	200	159-A1

© 2019 Feldman WB et al. *JAMA Network Open.*

Question	Trial	# Asked	Document
If you experienced a cardiac arrest and had about a one in ten chance of surviving, would you be willing to be part of this study without first providing consent?	PART[14]	303	172-A1
	PART[14]	400	173-A1
If you were severely injured and bleeding and were being treated by the paramedics in your community, would you want to be enrolled in this type of study?	HS-TBI[1]/ HS-Shock[2]	59	Draft 366-A1
	PolyHeme[6]	6	60-A1
	PolyHeme[6]	15	87-A1
	PolyHeme[6]	18	88-A1
	PolyHeme[6]	25	88-A1
	PolyHeme[6]	17	88-A1
	PolyHeme[6]	134	92-A1
	PolyHeme[6]	10	105-A1
	PolyHeme[6]	4	Draft 92-A1
	PolyHeme[6]	1	Draft 93-A1
	PolyHeme[6]	2	Draft 95-A1
	PolyHeme[6]	10	Draft 95-A1
	PolyHeme[6]	12	Draft 95-A1
	PolyHeme[6]	9	Draft 95-A1
	PolyHeme[6]	24	Draft 95-A1
	PolyHeme[6]	1	Draft 107-A1
	PolyHeme[6]	1	Draft 107-A1
	PolyHeme[6]	11	Draft 110-A1
	PolyHeme[6]	11	Draft 110-A1
	PolyHeme[6]	21	Draft 110-A1
	PolyHeme[6]	6	Draft 127-A1
	PolyHeme[6]	1	Draft 179-A1
	PolyHeme[6]	3	Draft 179-A1
	PolyHeme[6]	13	Draft 179-A1
	PolyHeme[6]	16	Draft 179-A1
	PolyHeme[6]	55	Draft 196-A1
	PolyHeme[6]	3	Draft 196-A1
	PolyHeme[6]	13	Draft 196-A1
	PolyHeme[6]	3	Draft 196-A1
	PolyHeme[6]	35	Draft 276-A1
	PolyHeme[6]	35	Draft 281-A1
	PolyHeme[6]	17	Draft 287-A1
	PolyHeme[6]	26	Draft 287-A1
	PolyHeme[6]	13	Draft 291-A1
	PolyHeme[6]	64	Draft 291-A1
	PolyHeme[6]	41	Draft 310-A1
	PolyHeme[6]	9	Draft 310-A1
	PolyHeme[6]	118	Draft 310-A1
	PolyHeme[6]	9	Draft 311-A1
	PolyHeme[6]	21	Draft 311-A1
	PolyHeme[6]	2	Draft 311-A1
	PolyHeme[6]	9	Draft 311-A1
If you were severely injured and bleeding and were being treated by the paramedics in your community, would you wish to be excluded?	PolyHeme[6]	4	93-A1
	PolyHeme[6]	5	93-A1
	PolyHeme[6]	4	93-A1
	PolyHeme[6]	5	93-A1
	PolyHeme[6]	50	93-A1

© 2019 Feldman WB et al. *JAMA Network Open.*

Question	Trial	# Asked	Document
Would you be willing to participate in this study or have a family member participate without your consent?	MERCI[15]	180	Draft 81-A1
	MERCI[15]	192	Draft 81-A1
Based on the information presented I would be willing to participate in this research study should I become critically injured.	PolyHeme[6]	602	Draft 159-A1
If you or a loved one were to be severely injured and bleeding would you be willing to have a trained person start giving a blood substitute at the scene as part of a study to assess potential survival benefit without obtaining consent?	PolyHeme[6]	117	Draft 304-A1
If you were injured, would you want to be put in this type of study?	PolyHeme[6]	19	Draft 309-A1
Would you be willing to participate in the study if you were to have a head injury?	Epo Severe TBI[16]	295	Draft 353-A1
I would personally be willing to be enrolled in the study.	HYPO RESUS[9]	245	123-A1
	ALPS[11]	322	117-A1
	PART[14]	89	172-A1
	PART[14]	48	172-A1
If you had a traumatic brain injury and no family member could be found to make decisions for you, you would be okay with being included in the ProTECT study without consent.	ProTECT[17]	7,315	166-A1
If you were involved in a major trauma event and needed many blood transfusions, would you want to be enrolled in this type of study?	PROPPR[12]	138	130-A4
If you were unconscious after a major accident and needed a large amount of blood, would you accept being enrolled in this type of study, even though you couldn't provide consent?	PROPPR[12]	207	141-A1
I would be willing to participate in RAMPART if I were having a serious seizure.	RAMPART[18]	1,750	137-A2
I think it would be acceptable for me to be enrolled in this study without my written consent if I had a traumatic injury resulting in blood loss and my legal representative (spouse, children, guardian) could not be contacted.	AVERT[19]	309	132-A2
If you developed a seizure that would not stop, you would be okay with being included in ESETT without giving your consent ahead of time.	ESETT[20]	7,186	482-A1
Question not provided			
Willing for family/self to participate?	NABIS:HIIR[21]	14	119-A2
	NABIS:HIIR[21]	8	119-A2
	NABIS:HIIR[21]	12	119-A2
	NABIS:HIIR[21]	25	119-A2
	NABIS:HIIR[21]	25	119-A2
	NABIS:HIIR[21]	11	119-A2
	NABIS:HIIR[21]	23	119-A2
	NABIS:HIIR[21]	14	119-A2
	NABIS:HIIR[21]	25	119-A2
	NABIS:HIIR[21]	5	119-A2
	NABIS:HIIR[21]	28	119-A2
Would want the experimental treatment knowing that it would be done without consent.	Prehospital Hypothermia[8]	57	106-A5

Question	Trial	# Asked	Document
	Prehospital Hypothermia[8]	90	106-A6
	Prehospital Hypothermia[8]	70	106-A7
…found it acceptable for themselves to be part of the study without consent.	PROPPR[12]	11	125-A1
**Total**		**34,671**	

*When more than 1 question is recorded in a single box, the answers to the last question is the one used for data abstraction. The first question in these instances is only provided to contextualize the second question.

© 2019 Feldman WB et al. *JAMA Network Open.*

eTable 2. Questions About Family Members*

Question	Trial	# Asked	Document
If one of your family members was severely injured and had a one in three chance of dying with standard treatment, would you want them to be entered into the study and possibly receive this experimental treatment, even if they or you couldn't give consent?	HS-TBI[1]/ HS-Shock[2]	502	2-A1
	HS-TBI[1]/ HS-Shock[2]	504	102-A1
	HS-TBI[1]/ HS-Shock[2]	502	Draft 358-A1
	HS-TBI[1]/ HS-Shock[2]	9	102-A1
	HS-TBI[1]/ HS-Shock[2]	73	102-A1
	HS-TBI[1]/ HS-Shock[2]	50	102-A1
	HS-TBI[1]/ HS-Shock[2]	2	102-A1
	HS-TBI[1]/ HS-Shock[2]	1	Draft 359-A1
	HS-TBI[1]/ HS-Shock[2]	2	Draft 359-A1
	HS-TBI[1]/ HS-Shock[2]	3	Draft 359-A1
If one of your family members was severely injured and had a one in three chance of not surviving with the standard treatment, would you want this experimental treatment given to them without written consent?	HS-TBI[1]/ HS-Shock[2]	186	Draft 361-A1
If one of your family members was severely injured as described, would you be willing for him or her to be part of the study without consent? This would include possibly being given the study fluid without consent.	HS-TBI[1]/ HS-Shock[2]	86	Draft 361-A1
If one of your family members suffered a cardiac arrest and had less than a 10% chance of surviving with standard treatment, would you want them to be entered into the study and possibly receive the experimental treatments, even if they or you couldn't give consent?	PRIMED[3,4]	502	2-A1
	PRIMED[3,4]	1	Draft 359-A1
	PRIMED[3,4]	2	Draft 359-A1
	PRIMED[3,4]	3	Draft 359-A1
If a family member of yours had a cardiac arrest and were treated by Seattle paramedics, would you want him or her to be enrolled into this type of study?	Mg-Diazepam-Both[7]	35	101-A1
	Mg-Diazepam-Both[7]	6	101-A1
	Mg-Diazepam-Both[7]	7	101-A1
If a family member of yours were severely injured and bleeding and were to be treated by the paramedics, would you want him or her to be enrolled in this type of study?	HS-TBI[1]/ HS-Shock[2]	59	Draft 366-A1
	PolyHeme[6]	6	60-A1
	PolyHeme[6]	15	87-A1
	PolyHeme[6]	18	88-A1
	PolyHeme[6]	25	88-A1
	PolyHeme[6]	17	88-A1
	PolyHeme[6]	134	92-A1
	PolyHeme[6]	10	105-A1
	PolyHeme[6]	4	Draft 92-A1

© 2019 Feldman WB et al. *JAMA Network Open.*

Question	Trial	# Asked	Document
	PolyHeme[6]	1	Draft 93-A1
	PolyHeme[6]	2	Draft 95-A1
	PolyHeme[6]	10	Draft 95-A1
	PolyHeme[6]	12	Draft 95-A1
	PolyHeme[6]	9	Draft 95-A1
	PolyHeme[6]	24	Draft 95-A1
	PolyHeme[6]	1	Draft 107-A1
	PolyHeme[6]	1	Draft 107-A1
	PolyHeme[6]	11	Draft 110-A1
	PolyHeme[6]	11	Draft 110-A1
	PolyHeme[6]	21	Draft 110-A1
	PolyHeme[6]	6	Draft 127-A1
	PolyHeme[6]	55	Draft 196-A1
	PolyHeme[6]	3	Draft 196-A1
	PolyHeme[6]	13	Draft 196-A1
	PolyHeme[6]	3	Draft 196-A1
	PolyHeme[6]	35	Draft 276-A1
	PolyHeme[6]	35	Draft 281-A1
	PolyHeme[6]	17	Draft 287-A1
	PolyHeme[6]	26	Draft 287-A1
	PolyHeme[6]	13	Draft 291-A1
	PolyHeme[6]	64	Draft 291-A1
	PolyHeme[6]	41	Draft 310-A1
	PolyHeme[6]	9	Draft 310-A1
	PolyHeme[6]	118	Draft 310-A1
	PolyHeme[6]	9	Draft 311-A1
	PolyHeme[6]	21	Draft 311-A1
	PolyHeme[6]	2	Draft 311-A1
	PolyHeme[6]	9	Draft 311-A1
If a family member of your family were severely injured and bleeding and were to be treated by the paramedics, would you want him or her to be enrolled in this type of study?	PolyHeme[6]	1	Draft 179-A1
	PolyHeme[6]	3	Draft 179-A1
	PolyHeme[6]	13	Draft 179-A1
	PolyHeme[6]	16	Draft 179-A1
If a family member of yours were severely injured and bleeding and were to be treated by the paramedics, would you want him or her to be excluded from this study?	PolyHeme[6]	4	93-A1
	PolyHeme[6]	5	93-A1
	PolyHeme[6]	4	93-A1
	PolyHeme[6]	5	93-A1
	PolyHeme[6]	50	93-A1
If a family member were injured, would you want him/her in the study?	PolyHeme[6]	19	Draft 309-A1
I think it would be acceptable to enroll my family members into this study without consent if they had a serious traumatic brain injury and I could not be contacted.	ProTECT[17]	2,201	166-A1
If a close friend or family member of yours was involved in a major traumatic event requiring a massive blood transfusion, would you want him/her to be enrolled in this type of study?	PROPPR[12]	138	130-A4
If a close friend or family member of yours was involved in a major accident and needed a large amount of blood, would you want them to be enrolled in this type of study?	PROPPR[12]	207	141-A1
I would want a family member enrolled in RAMPART if they were having a seizure that would not stop on its own.	RAMPART[18]	96	137-A2

© 2019 Feldman WB et al. *JAMA Network Open.*

Question	Trial	# Asked	Document
I think it would be acceptable to enroll my family members in this study without consent if they had a traumatic injury resulting in blood loss and I could not be contacted.	AVERT[19]	309	132-A2
Question not provided			
…found it acceptable for a family member to be part of the study without consent.	PROPPR[12]	11	125-A1
**Total**		**6,428**	

*We exclude questions about whether individuals would be willing to give consent for their family members to participate in studies. The question is whether individuals would be willing to have family members enrolled into these types of studies without their consent. We also excluded questions directed at parents about enrollment of their own children in pediatric studies.

© 2019 Feldman WB et al. *JAMA Network Open.*

eTable 3. Questions About Community

Question	Trial	# Asked	Document
Would you support such a study being done in your community, specifically, a study in which patients and their families do not have a chance to give their consent to be in the study?	Mg-Diazepam-Both[7]	35	101-A1
	Mg-Diazepam-Both[7]	6	101-A1
	Mg-Diazepam-Both[7]	7	101-A1
Are you in favor of the researchers carrying out this study in your community?	CCC[10]	503	114-A1
	ALPS[11]	503	114-A1
Are you in favor of or opposed to the researchers carrying out this study in your community?	ALPS[11]	500	119-A1
	ALPS[11]	252	120-A1
	ALPS[11]	250	135-A1
	ALPS[11]	502	138-A1
	ALPS[11]	500	151-A1
Is it acceptable to you for this research to be conducted in your community?	PROPPR[12]	500	130-A2
	PROPPR[12]	252	136-A1
	PROPPR[12]	207	141-A1
Do you believe the research is in the best interest of the patients and community?	PROPPR[12]	500	130-A3
How would you react to the following statement…"I think it is acceptable for this study to be performed in my community."	Pediatric Lorazepam[22]	508	127-A10
Are you in favor or not in favor of this study being conducted in your community?	PART[14]	400	173-A1
	ESETT[20]	1,160	482-A1
Would you support a study such as the one described at this meeting being conducted in this community, specifically, a study in which severely injured and bleeding patients would be enrolled without giving their informed consent?	HS-TBI[1]/ HS-Shock[2]	59	Draft 366-A1
	PolyHeme[6]	6	60-A1
	PolyHeme[6]	15	87-A1
	PolyHeme[6]	18	88-A1
	PolyHeme[6]	25	88-A1
	PolyHeme[6]	17	88-A1
	PolyHeme[6]	134	92-A1
	PolyHeme[6]	4	93-A1
	PolyHeme[6]	5	93-A1
	PolyHeme[6]	4	93-A1
	PolyHeme[6]	5	93-A1
	PolyHeme[6]	50	93-A1
	PolyHeme[6]	4	Draft 92-A1
	PolyHeme[6]	1	Draft 93-A1
	PolyHeme[6]	2	Draft 95-A1
	PolyHeme[6]	10	Draft 95-A1
	PolyHeme[6]	12	Draft 95-A1
	PolyHeme[6]	9	Draft 95-A1
	PolyHeme[6]	24	Draft 95-A1
	PolyHeme[6]	1	Draft 107-A1
	PolyHeme[6]	1	Draft 107-A1
	PolyHeme[6]	11	Draft 110-A1
	PolyHeme[6]	11	Draft 110-A1
	PolyHeme[6]	21	Draft 110-A1

© 2019 Feldman WB et al. *JAMA Network Open.*

Question	Trial	# Asked	Document
	PolyHeme[6]	6	Draft 127-A1
	PolyHeme[6]	1	Draft 179-A1
	PolyHeme[6]	3	Draft 179-A1
	PolyHeme[6]	13	Draft 179-A1
	PolyHeme[6]	16	Draft 179-A1
	PolyHeme[6]	55	Draft 196-A1
	PolyHeme[6]	3	Draft 196-A1
	PolyHeme[6]	13	Draft 196-A1
	PolyHeme[6]	3	Draft 196-A1
	PolyHeme[6]	35	Draft 281-A1
	PolyHeme[6]	13	Draft 291-A1
	PolyHeme[6]	41	Draft 310-A1
	PolyHeme[6]	9	Draft 310-A1
	PolyHeme[6]	118	Draft 310-A1
	PolyHeme[6]	9	Draft 311-A1
	PolyHeme[6]	21	Draft 311-A1
	PolyHeme[6]	2	Draft 311-A1
	PolyHeme[6]	9	Draft 311-A1
In view of what you have learned here, are you willing for this study to be done in your community?	MERCI[15]	180	Draft 81-A1
	MERCI[15]	192	Draft 81-A1
Would you be in favor of a study such as this being conducted in this community, specifically, a study in which severely injured and bleeding patients would be enrolled without giving their informed consent?	PolyHeme[6]	35	Draft 276-A1
	PolyHeme[6]	64	Draft 291-A1
Would you support conducting a study such as the one described in this community, specifically, a study in which severely injured and bleeding patients would be enrolled without giving their informed consent?	PolyHeme[6]	17	Draft 287-A1
Would you support conducting a study such as the one described in this community meeting, specifically a study in which severely injured and bleeding patients would be enrolled without giving their informed consent?	PolyHeme[6]	26	Draft 287-A1
Would you like to see this type of study being conducted with our hospital and EMS units as well as other medical centers to help determine if using a blood substitute improves survival after severe injury with bleeding?	PolyHeme[6]	117	Draft 304-A1
Are you willing for this study to be done in your community?	Epo Severe TBI[16]	295	Draft 353-A1
I support the clinical trial taking place in our community.	ResQPro[23]	139	121
Do you think this study should be conducted in your community?	ProTECT[17]	1,736	166-A1
Will you allow us to conduct a research study in this community of people experiencing a major traumatic event requiring multiple blood transfusions who are unable to give their own informed consent?	PROPPR[12]	138	130-A4
Do you support this study being done in your community?	RAMPART[18]	3,280	137-A2
I think it is acceptable for this study to be performed in my community.	AVERT[19]	309	132-A2
Question not provided			
Willing for community to participate?	NABIS:HIIR[21]	14	119-A2
	NABIS:HIIR[21]	8	119-A2

Question	Trial	# Asked	Document
	NABIS:HIIR[21]	12	119-A2
	NABIS:HIIR[21]	25	119-A2
	NABIS:HIIR[21]	25	119-A2
	NABIS:HIIR[21]	11	119-A2
	NABIS:HIIR[21]	23	119-A2
	NABIS:HIIR[21]	14	119-A2
	NABIS:HIIR[21]	25	119-A2
	NABIS:HIIR[21]	5	119-A2
	NABIS:HIIR[21]	28	119-A2
Willing to allow study in community ("approval")?	Polyheme[6]	145	75-A1
**Total**		**14,267**	

© 2019 Feldman WB et al. *JAMA Network Open.*

eTable 4. Questions About Exceptions from Informed Consent in General

Question	Trial	# Asked	Document
Do you believe that this exception to written consent is justified and in the best interest of the patients?	HS-TBI[1]/ HS-Shock[2]	150	15-A1
Do you believe that this exception to written consent is justified and in the best interests of the patients and community or not?	HS-TBI[1]/ HS-Shock[2]	302	15-A1
	HS-TBI[1]/ HS-Shock[2]	305	20-A1
	HS-TBI[1]/ HS-Shock[2]	500	Draft 357-A1
	HS-TBI[1]/ HS-Shock[2]	502	Draft 358-A1
	Hu23F2G	501	39-A4
Do you believe that this exception to written consent is justified and in the best interests of the patients and community?	HS-TBI[1]/ HS-Shock[2]	505	Draft 366-A1
	HS-TBI[1]/ HS-Shock[2]	5	Draft 366-A1
	PolyHeme[6]	500	86
Do you believe that this exception to written consent is justified in a research study of a new drug for treating patients who have been severely injured?	Hu23F2G[5]	508	39-A6
Do you think that it is all right to do this study when prior consent is not possible?	Prehospital Hypothermia[8]	100	106-A2
Do you believe that this exception to written consent before enrolling a patient into this study is justified?	PROPPR[12]	500	130-A3
Do you think anyone should be enrolled in a study without their consent?	PROPPR[12]	500	131-A1
How would you react to the following statement…"Involving patients in a medical research study without asking their permission first is acceptable in emergency circumstances."	Pediatric Lorazepam[22]	508	127-A10
Would you support a study with life-saving potential in which severely injured and bleeding patients would be enrolled without giving their informed consent?	PolyHeme[6]	10	105-A1
Do you feel it is acceptable to conduct studies using exception from informed consent?	PolyHeme[6]	9	Draft 401
The benefits of the research (learning the best treatment for traumatic injury) justify doing a study when consent cannot be obtained.	HYPO RESUS[9]	245	123-A1
The benefits of the research (learning the best treatment for cardiac arrest) justify doing a study when consent cannot be obtained.	ALPS[11]	322	117-A1
Sometimes no family member can be found to make medical decisions for patients with traumatic injury. It is okay to include those patients in the ProTECT study without consent?	ProTECT[17]	7,241	166-A1
Involving patients in a medical research study without asking their permission is acceptable in emergency situations.	RAMPART[18]	3,200	137-A2
The benefits of the research (learning the best treatment for cardiac arrest) justify doing a study when consent cannot be obtained.	PART[14]	89	172-A1
	PART[14]	48	172-A1
I think that it is acceptable for someone having a seizure that will not stop to be enrolled in ESETT, even if they have not given advanced permission.	ESETT[20]	300	482-A1

© 2019 Feldman WB et al. *JAMA Network Open.*

Question	Trial	# Asked	Document
Question not provided			
Object to enrollment of subjects without prior consent.	PolyHeme[6]	145	75-A1
**Total**		**16,995**	

© 2019 Feldman WB et al. *JAMA Network Open.*

eReferences

1. Bulger EM, May S, Brasel KJ, et al; ROC Investigators. Out-of-hospital hypertonic resuscitation following severe traumatic brain injury: a randomized controlled trial. *JAMA*. 2010;304(13):1455-1464.
2. Bulger EM, May S, Kerby JD, et al; ROC Investigators. Out-of-hospital hypertonic resuscitation after traumatic hypovolemic shock: a randomized, placebo controlled trial. *Ann Surg*. 2011;253(3):431-441.
3. Aufderheide TP, Nichol G, Rea TD, et al; ROC Investigators. A trial of an impedance threshold device in out-of-hospital cardiac arrest. *N Engl J Med*. 2011;365(9):798-806.
4. Stiell IG, Nichol G, Leroux BG, et al; ROC Investigators. Early versus later rhythm analysis in patients with out-of-hospital cardiac arrest. *N Engl J Med*. 2011;365(9):787-797.
5. Regulations.gov. ICOS Corporation, community consultation and public disclosure information for waiver of informed consent, BB IND-7371, public disclosure information, report 3. https://www.regulations.gov/document?D=FDA-1995-S-0036-0039. Accessed May 29, 2019.
6. Moore EE, Moore FA, Fabian TC, et al; PolyHeme Study Group. Human polymerized hemoglobin for the treatment of hemorrhagic shock when blood is unavailable: the USA multicenter trial. *J Am Coll Surg*. 2009;208(1):1-13.
7. Longstreth WT, Fahrenbruch CE, Olsufka M, Walsh TR, Copass MK, Cobb LA. Randomized clinical trial of magnesium, diazepam, or both after out-of-hospital

© 2019 Feldman WB et al. *JAMA Network Open.*

cardiac arrest. *Neurology*. 2002;59(4):506-514.

8. Kim F, Nichol G, Maynard C, et al. Effect of prehospital induction of mild hypothermia on survival and neurological status among adults with cardiac arrest: a randomized clinical trial. *JAMA*. 2014;311(1):45-52.
9. Schreiber MA, Meier EN, Tisherman SA, et al; ROC Investigators. A controlled resuscitation strategy is feasible and safe in hypotensive trauma patients: results of a prospective randomized pilot trial. *J Trauma Acute Care Surg*. 2015;78(4):687-695.
10. Nichol G, Leroux B, Wang H, et al; ROC Investigators. Trial of continuous or interrupted chest compressions during CPR. *N Engl J Med*. 2015;373(23):2203-2214.
11. Kudenchuk PJ, Brown SP, Daya M, et al; ROC Investigators. Amiodarone, lidocaine, or placebo in out-of-hospital cardiac arrest. *N Engl J Med*. 2016;374(18):1711-1722.
12. Holcomb JB, Tilley BC, Baraniuk S, et al; PROPPR Study Group. Transfusion of plasma, platelets, and red blood cells in a 1:1:1 vs a 1:1:2 ratio and mortality in patients with severe trauma: the PROPPR randomized clinical trial. *JAMA*. 2015;313(5):471-482.
13. US National Library of Medicine. Prehospital tranexamic acid use for traumatic brain injury. https://clinicaltrials.gov/ct2/show/NCT01990768. Accessed May 29, 2019.
14. Wang HE, Schmicker RH, Daya MR, et al. Effect of a strategy of initial laryngeal tube insertion vs endotracheal intubation on 72-hour survival in adults with out-of-

© 2019 Feldman WB et al. *JAMA Network Open.*

hospital cardiac arrest: a randomized clinical trial. *JAMA*. 2018;320(8):769-778.

15. Smith WS, Sung G, Saver J, et al; Multi MERCI Investigators. Mechanical thrombectomy for acute ischemic stroke: final results of the multi MERCI trial. *Stroke*. 2008;39(4):1205-1212.

16. Robertson CS, Hannay HJ, Yamal J-M, et al; Epo Severe TBI Trial Investigators. Effect of erythropoietin and transfusion threshold on neurological recovery after traumatic brain injury: a randomized clinical trial. *JAMA*. 2014;312(1):36-47.

17. Wright DW, Yeatts SD, Silbergleit R, et al; NETT Investigators. Very early administration of progesterone for acute traumatic brain injury. *N Engl J Med*. 2014;371(26):2457-2466.

18. Silbergleit R, Durkalski V, Lowenstein D, et al; NETT Investigators. Intramuscular versus intravenous therapy for prehospital status epilepticus. *N Engl J Med*. 2012;366(7):591-600.

19. US National Library of Medicine. AVERT Shock: arginine vasopressin during the early resuscitation of traumatic shock. https://clinicaltrials.gov/ct2/show/NCT01611935. Accessed May 29, 2019.

20. US National Library of Medicine. A multicenter, randomized, blinded, comparative effectiveness study of fosphenytoin, valproic acid, or levetiracetam in the emergency department treatment of patients with benzodiazepine-refractory status epilepticus. https://clinicaltrials.gov/ct2/show/NCT01960075. Accessed May 29, 2019.

21. Clifton GL, Valadka A, Zygun D, et al. Very early hypothermia induction in patients with severe brain injury (the National Acute Brain Injury Study:

Hypothermia II): a randomised trial. *Lancet Neurol*. 2011;10(2):131-139.

22. Chamberlain JM, Okada P, Holsti M, et al; Pediatric Emergency Care Applied Research Network (PECARN). Lorazepam vs diazepam for pediatric status epilepticus: a randomized clinical trial. *JAMA*. 2014;311(16):1652-1660.

23. Raubenolt A, Mastenbrook J, Franklin K, Fales W. Abstracts for the 2014 NAEMSP Scientific Assembly: comparison of standard CPR versus CPR with an intrathoracic pressure regulator versus active compression decompression CPR plus an impedance threshold device during out-of-hospital cardiac arrest. 2014 *Prehospital Emergency Care*. 2014;18(1):123-162.

© 2019 Feldman WB et al. *JAMA Network Open.*
